# Does the format matter? A cross-sectional analysis of suspected injuries and game events across the different versions of field hockey

**DOI:** 10.3389/fspor.2025.1565036

**Published:** 2025-09-02

**Authors:** S. W. West, J. Bovington, J. Dale, T. Alexander, A. Keogh, S. Holden

**Affiliations:** ^1^ Centre for Health, and Injury and Illness Prevention in Sport, University of Bath, Bath, United Kingdom; ^2^ UK Collaborating Centre on Injury and Illness Prevention in Sport, University of Bath, Bath, United Kingdom; ^3^ Sport Injury Prevention Research Centre, Faculty of Kinesiology, University of Calgary, Calgary, Alberta, Canada; ^4^ Department of Health, University of Bath, Bath, United Kingdom; ^5^ Der Club an der Alster, Hamburg, Germany; ^6^ School of Medicine, Trinity College Dublin, Dublin 2, Ireland; ^7^ Institute for Sport and Health, University College Dublin, Dublin, Ireland; ^8^ School of Public Health, Physiotherapy and Sports Science, University College Dublin, Dublin, Ireland

**Keywords:** policy, prevention, sport, injury, performance

## Abstract

**Introduction:**

Field Hockey is a popular global sport played by both men and women in three different formats: 11-a-side outdoor hockey (11s), 6-a-side indoor hockey, and 5-a-side Hockey5s. To date, comparisons across formats for match events and injury rates have not occurred.

**Methods:**

Using an established video analysis methodology, this study aimed to compare match events (per 10 min of play) and suspected injury rates across formats and genders. A hockey-specific video coding window was co-created with community partners, before being deployed to capture outcomes of interest in 30 international hockey matches (10 per format, 50% male/female).

**Results:**

Twenty-seven suspected injuries were identified. The most common trends in these injuries included; being to the head/neck (26%); contusion in nature (74%); ball-player contact mechanism (44%); 74% to defending player. No evidence of significant differences in injury rates between formats or genders were identified, however a trend towards higher rates in men's vs. women's was identified [Rate ratio (RR) range: 1.14–5.00] as well as in Hockey5s for men and 11s for women. Game events differed significantly across formats for both men and women. Increased outcomes which could be deemed “exciting” (e.g., shots, shooting zone entries) differed between formats, however the success (e.g., shots on target vs. off target) of these increased “exciting” outcomes was often lower in formats with higher rates.

**Discussion:**

The findings of this study suggest the need for a more in-depth investigation into differences between formats, which may include mixed methods approaches to capture fan engagement, player perception, and injury risk.

## Introduction

Field Hockey (hockey hereafter) is one of the world's most popular team sports, played by both men and women. The main formats include outdoor 11-a-side (outdoor), indoor 5-a-side (indoor) and Hockey5s. Outdoor hockey is the traditional 11-a-side format, which has been played in the Olympic Games since 1908. Indoor Hockey's first introduction to the global stage was in the 2003 Indoor World Cup and most recently Hockey5s was introduced in 2014 at the Youth Olympic Games, before the first World Cup in 2024 (1). There are distinct differences between formats including the rules, number of players, number of substitutes, match duration, pitch dimensions, boundary configurations and scoring locations (Supplementary Table S1).

As the most common format of hockey, outdoor hockey has the largest evidence on injury epidemiology and risk within the game. Despite this, significant heterogeneity exists in the injury definitions used, the methodologies used to capture injury data, and the reporting processes (2). This limits our understanding as to the risk of injury within the game, especially across the various formats of the sport. Theilen et al. (3) have reported match injury rates of international outdoor hockey ranging from 23.4–44.2/1,000 h in the women's game and 20.8–90.9/1,000 h in the men's game. Literature within the indoor format is sparse in nature, with one study suggesting a match injury rate of 17.1/1,000 h [Confidence Intervals (CI): 3.4–30.7] in women and 5.2 (CI: 1.3–9.0) in men (4). In the interests of growing access to the sport and promoting an exciting and compelling viewing experience, modification of the traditional formats of indoor and outdoor hockey has led to the development of Hockey5s. Hockey5s lacks data on injury rates, but it is presented as a highly demanding and intense version of the sport, with very short intervals for recovery (5). Given the differences in the rules and nature of the sport across each of the hockey formats, the potential for different injury rates and epidemiological characteristics exists. No studies have investigated differences in match events and suspected injury outcomes across formats.

The use of video analysis in sport to understand game demands (6), injury risk (7, 8), injury mechanisms (7–9) and use of prevention equipment (10) has grown in recent years and provides opportunity to compare across formats. Despite the known limitations of such investigations (11), studies using video can inform injury prevention priorities and evaluations in a multi-faceted approach, particularly when paired with validated surveillance methodologies. Using video analysis, Theilen et al. (8) have reported match injury rates for 11-a-side hockey of 34.2/1,000 h (CI: 14.0–51.3) for women and 57.9 (29.8–73.1) for men, with injuries most often occurring in the circle, most commonly leading to contusions (91%: women; 96%: men) and most commonly to the head/face (33%: women; 30%: men).

Therefore, the aim of the this study is to investigate and compare the game events, injury incidence, and suspected injury characteristics across three hockey formats (both men and women) using validated methodology (7).

## Methods

### Setting and population

This is a cross-sectional study using publicly available video footage from online sources. Footage from 2022 or later was considered eligible, to ensure the reflected contemporary trends within the game. Only International games were included, to standardise level of play across formats. The footage was of broadcast quality, often including replays of key events, although this was beyond the control of the research team. Games were selected based on online availability (i.e., YouTube), and no information related to individual players or teams was captured to maintain anonymity of players. This study was approved in line with the University of Bath Ethics Committee (Ref: 1553-1276).

### Procedures

Game footage was downloaded and stored as video files on a secure University server. Videos were analysed using Nacsport Video Analysis software (Scout Plus V 6.5.0, NACSPORT, S.L). To develop a coding window to identify injury and game behaviours, a bespoke video coding template was co-created to capture all relevant outcomes for the study with the support of several community partners. This group was made up of five men and three women who held one or more of the following roles: five researchers, two video analysts, two international players, one recreational player, one international umpire, and one coach with experience from amateur to professional, primarily in 11-a-side outdoor and indoor formats. An initial consultation with each community partner was held to identify key concepts or aspects which the templates required to include (e.g., in the absence of a formal definition, aspects related to “excitement” within the game were discussed between partners and operationalised as number of shots taken, number of entries into the shooting area, or number of counter attacks). Following this, the primary research team (SW, JB, JD) drafted a template which was circulated to the group for feedback. Clear operational definitions were created for each variable included in the coding window, following consultation with the group (Supplementary Appendix A). After formal consultation, the template was pilot tested, and adjustments made to optimise performance and avoid duplication (e.g., the inclusion of an unknown button for when footage was not of high enough quality to be able to accurately provide the answer). The final template is available as Supplementary Appendix B and includes actions such as passes, tackles, shots, shooting zone entries, and fouls. Descriptions of these actions were also included (e.g., pass type, pass success, shot success, shot type). Other variables indicating high-risk actions were also captured including shot height, and the number of players which shots pass through. Following this an inter-rater reliability assessment was undertaken for each of the coders (JB, JD) with the lead author acting as gold standard, having extensive experience using video analysis software for this purpose and a background in hockey. All coders were required to meet an average reliability threshold of >80% agreement with the gold standard before coding games could begin. In cases where the 80% threshold was not met, a retraining process was undertaken to clarify any potentially subjective coding variables. The inter-rater reliability process was undertaken on one half of hockey for each of the three formats to ensure there were not format-specific challenges. One month after initial coding, intra-rater reliability was also conducted with all coders achieving >90% agreement. During game coding, footage could be slowed down and watched multiple times to obtain a final decision.

Injuries were captured using previously developed suspected injury criteria for football (12) and validated for use in rugby union (7) by non-medically trained coders. Suspected injuries were defined as meeting at least one of four criteria which included: The game is stopped by the umpire for a player in distress; a player receives medical attention; a player remains down for >10 s; a player appears to be in pain caused by an inciting event. Suspected concussions were identified using the criteria validated by West et al. (7) which includes the criteria of video signs of concussion (13).

### Statistical analysis

Match event rates were calculated as a rate per 10 min of play (due to differences in game length across formats). Match event rates were calculated for tackles, passes, turnovers, counterattacks, shots, entries into the shooting zone, and fouls. Significant differences between formats and genders were conservatively estimated when 95% confidence intervals (CI) did not overlap. Rate ratios were calculated using univariate Poisson regression for any comparison where CI did not overlap, with significance was set at an alpha of <0.05*.* Game event characteristics were described using proportions. Suspected injury and concussion rates were calculated per 1,000 h, and rate ratios were calculated to determine significant differences between formats and genders. Descriptive statistics were used to outline the epidemiological characteristics of the injuries. All data cleaning, formatting and analysis was undertaken in STATA (StataCorp, 2021, Stata Statistical Software: Release 17. College Station, TX: StataCorp LLC).

### Patient and public involvement statement

Both players and members of the hockey community (i.e., coaches, umpires, clinicians, and analysts) were included in the design and interpretation of the study.

## Results

A convenience sample of 30 games (including invitational international series and Continental competitions) were used for analysis which comprised of five games from each format for men and five for women. This led to a total exposure time of 220 h of outdoor hockey, 79 h of indoor and 50 h of Hockey5s.

### Suspected injury and suspected concussion

In total there were 27 suspected injuries identified [15 from outdoor (8 men, 7 women), 6 from indoor (5 men, 1 women) and 6 Hockey5s (5 men, 1 women) with 3 suspected concussions. Of the injury criteria, all suspected injuries showed a player in pain, 81% required the game to be stopped, 67% left a player down for >10 s, and 37% required medical attention.

**Figure 1:**
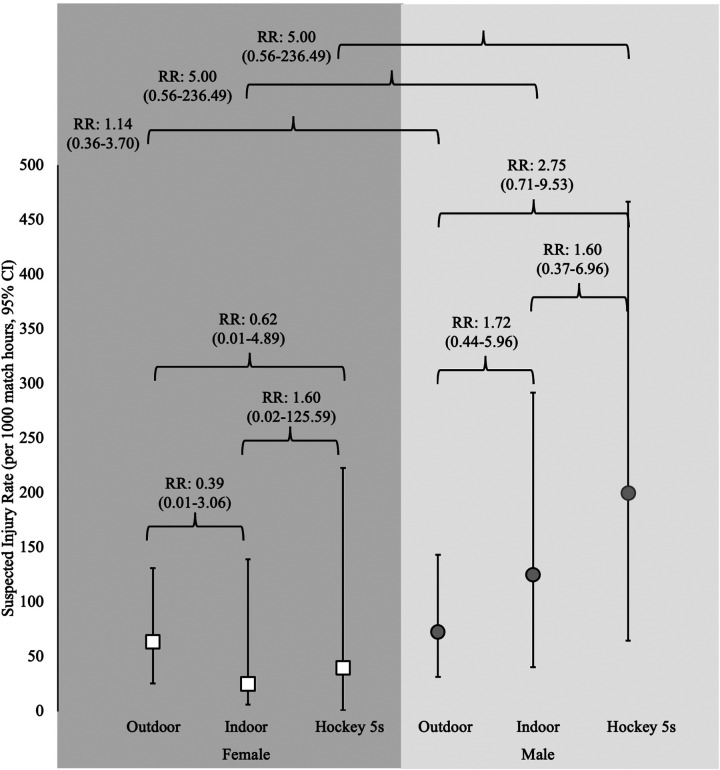
Injury incidence rates and rate ratios for each format of play and gender.

In all formats of the sport, the rates of injury were higher in the men's game vs. the women's game, however none of these differences were statistically significant (Figure 1, Supplementary Table S2). The rate of injury was highest in men's Hockey5s [200.0/1,000 h (95% CI: 64.9–466.7)] followed by men's indoor and outdoor (Figure 1, Supplementary Table S2). In the women's game, outdoor had the highest rate of injury [63.6/1,000 h (95% CI: 25.6–131.1)] followed by Hockey5s and Indoor (Figure 1, Supplementary Table S2). None of the differences in injury rates between formats were statistically significant.

Table 1 presents detail on all injuries identified in the study. The head/neck (26%) was the most commonly injured site. Seventy-four percent of injuries were contusions, 19% lacerations, and suspected muscle strain and suspected dislocation made up the remaining injury types (each 4%). Forty-four percent of suspected injuries were the result of ball-player contact, followed by stick-player (26%), player-player (22%), and player-turf or player-goal frame (4%). Specifically, the event associated with suspected injuries was a tackle (44%), blocking/ being hit (33%), short corner defense (11%) and ball carrying, player contact or illegal foul play (all 4%). Seventy percent of all injuries were to the defending player. The management of these suspected injuries demonstrated that only 37% led to removal from the game. The umpire penalized the offending player 78% of the times there was a suspected injury, with a free hit against them, however only one (4%) led to a temporary removal from the game (a two-minute green card suspension).

**Table 1:** Description of all suspected injuries across formats and genders.

Injury number	Format	Gender	Injury location	Injury event	Injury mechanism	Ball height	Phase of game	Injury type	Time in game	Location
1	Outdoor	Men	Head	Tackle	Ball-player contact	Deflection	Attacking	Laceration	2nd Half	Between 25s
2			Head	Blocking	Ball-player contact	Lifted	Defending	Laceration	2nd Half	Between 25s
3			Trunk	Tackle	Player-player contact	NA	Defending	Contusion	2nd Half	Circle
4			Trunk	Blocking	Ball-player contact	Lifted	Defending	Contusion	2nd Half	Inside 25
5			Hip	Interception	Ball-player contact	Lifted	Defending	Contusion	2nd Half	Inside 25
6			Leg	Penalty Corner	Ball-player contact	Lifted	Defending	Contusion	2nd Half	Circle
7			Arm	Tackle	Stick-player contact	NA	Defending	Contusion	2nd Half	Between 25s
8			Arm	Illegal Play	Player-player contact	NA	Attacking	Contusion	2nd Half	Circle
9		Women	Leg	Blocking	Stick-player contact	NA	Defending	Contusion	2nd Half	Circle
10			Leg	Player contact	Post-player contact	NA	Attacking	Contusion	2nd Half	Circle
11			Trunk	Penalty Corner	Ball-player contact	Lifted	Defending	Contusion	2nd Half	Circle
12			Head	Blocking	Ball-player contact	Deflection	Defending	Contusion	2nd Half	Inside 25
13			Hand	Carrying	Stick-player contact	NA	Attacking	Laceration	2nd Half	Between 25s
14			Head	Tackle	Ball-player contact	Deflection	Attacking	Laceration	2nd Half	Inside 25
15			Hand	Blocking	Ball-player contact	Flat	Defending	Contusion	2nd Half	Between 25s
16	Indoor	Men	Chest	Blocking	Ball-player contact	Deflection	Defending	Contusion	1st Half	Circle
17			Trunk	Penalty Corner	Ball-player contact	Lifted	Defending	Contusion	1st Half	Circle
18			Arm	Tackle	Player-player contact	NA	Defending	Contusion	2nd Half	Outside Circle
19			Head	Tackle	Stick-player contact	NA	Defending	Contusion	2nd Half	Outside Circle
20			Hand	Tackle	Player-player contact	NA	Attacking	Dislocation	2nd Half	Circle
21		Women	Leg	Tackle	Player-player contact	NA	Attacking	Contusion	2nd Half	Outside Circle
22	5s	Men	Leg	Blocking	Stick-player contact	NA	Defending	Contusion	1st Half	Midline to endline
23			Trunk	Tackle	Player-player contact	NA	Attacking	Contusion	1st Half	Midline to 23 m line
24			Head	Tackle	Stick-player contact	NA	Defending	Laceration	1st Half	Midline to endline
25			Head	Tackle	Stick-player contact	NA	Defending	Contusion	2nd Half	Midline to endline
26			Trunk	Blocking	Ball-player contact	Lifted	Defending	Contusion	1st Half	Midline to 23 m line
27		Women	Leg	Tackle	Player-turf contact	NA	Defending	Muscle Strain	1st Half	Circle

### Game events

The rates of all games events recorded are reported per 10 min of play in Table 2. In the men's games, there were significant differences in passing rates in Indoor (vs. outdoor and Hockey5s), turnover rates in Hockey5s (vs. outdoor), counterattack rates in Hockey5s (vs. outdoor), shots in Hockey5s (vs. indoor and outdoor), and shooting zone entries in Hockey5s (vs. outdoor: Table 2). In the women's game, there were significant differences in passing rates in Indoor (vs. outdoor and Hockey5s), counterattacks in indoor (vs. Hockey5s) and shots in Hockey5s (vs. indoor and outdoor: Table 2).

**Table 2:** Game event rates per 10 min of play in each respective format for men and women.

Event	Men			Women		
	Outdoor	Indoor	5s	Outdoor	Indoor	5s
Tackle	4.0 (2.4–6.1)	6.4 (4.4–9.0)	6.0 (4.0–8.6)	5.8 (3.9–8.3)	5.0 (3.2–7.4)	7.0 (4.9–9.7)
Pass	91.0 (82.8–99.8)	124.8 (115.2–135.0)	49.8 (43.8–56.4)	90.2 (82.1–98.9)	119.0 (109.6–129.0)	55.4 (49.1–62.3)
Turnover	19.4 (16.7–23.7)	27.2 (22.8–32.2)	28.2 (23.7–33.3)	25.6 (21.4–30.4)	30.6 (25.9–35.9)	33.4 (28.5–38.9)
Counterattack	0.4 (0.1–1.4)	1.8 (0.8–3.4)	4.6 (2.9–6.9)	3.2 (1.8–5.2)	5.2 (3.4–7.6)	1.8 (0.8–3.4)
Shots	2.6 (1.4–4.4)	7.2 (5.0–10.0)	20.4 (16.6–24.8)	2	5.6 (3.7–8.1)	15.2 (11.9–19.0)
Entry into shooting zone	10.8 (8.1–14.1)	18.4 (14.8–22.6)	24.8 (20.6–29.6)	6.8 (4.7–9.5)	14.4 (11.3–18.1)	25.2 (21.0–30.0)
Foul	11.4 (8.6–14.8)	11.8 (8.9–15.2)	13.0 (10.0–16.6)	14.0 (10.9–17.7)	16.6 (13.2–20.6)	19.4 (15.7–23.7)

Table 3 provides an outline of the descriptives associated with different events. Similarly, men's Hockey5s had the lowest proportion of on-target shots, while outdoor had the highest proportion of on-target shots. The proportion of shots leading to goals ranged from 13% in Hockey5s to 28% in outdoor for men and from 15% in outdoor and Hockey5s to 22% in indoor in women. In most cases, shots were hit through between 1 and 2 players in men's Hockey5s and in all formats of the women's game. Hockey5s was the sport where the greatest number of shots were hit at a potentially dangerous height (i.e., above the shin pad when passing the nearest player) for both men and women, with this being significantly more so in the men's than women's game. Most fouls led to free hits (men's range: 74.4–89.1; women's range: 77.0–94.0), followed by penalty corners/challenges and cases where advantage was played.

**Table 3:** Game event descriptions.

Event			Men			Women		
	Descriptor	Characteristics	Outdoor	Indoor	5s	Outdoor	Indoor	5s
Pass	Pass Success	Successful	88.1 (86.9–89.3)	90.1 (88.8–91.2)	87.2 (84.4–89.6)	76.0 (74.3–77.6)	82.9 (81.3–84.4)	75.9 (72.5–79.2)
		Mistrap	1.6 (1.2–2.2)	1.4 (1.0–1.9)	1.8 (0.9–3.1)	7.4 (6.4–8.4)	6.6 (5.6–7.6)	3.9 (2.6–5.7)
		Intercepted	6.0 (5.2–7.0)	2.6 (2.0–3.3)	9.8 (7.7–12.3)	16.4 (15.0–17.9)	10.4 (9.2–11.7)	20.4 (17.4–23.7)
	Pass Type	Aerial	5.8 (4.9–6.8)	–	<1	3.7 (3.1–4.6)	–	<1
		Flat	90.2 (86.8–93.7)	99.4 (99.0–99.6)	95.3 (93.4–96.8)	92.1 (91.0–93.1)	98.2 (97.6–98.7)	98.7 (97.6–99.5)
		Lifted	4.7 (4.0–5.6)	<1	3.5 (2.2–5.2)	2.7 (2.1–3.4)	<1	<1
		Deflection	<1	<1	<1	1.2 (0.8–1.7)	1.8 (1.3–2.4)	–
Counterattack	Counterattack Success	Successful	58.3 (27.6–84.8)	67.6 (50.2–82.0)	58.2 (44.1–71.3)	37.8 (28.1–48.4)	49.0 (38.9–59.1)	81.0 (58.1–94.6)
Shooting	Shot Success	On Target	66.6 (55.1–76.9)	62.0 (53.5–70.0)	45.4 (39.3–51.7)	46.1 (34.5–57.9)	57.1 (47.3–66.5)	52.2 (44.7–60.0)
		Off Target	25.6 (16.4–36.8)	21.8 (15.3–29.5)	31.3 (25.7–37.3)	34.2 (23.7–46.0)	22.3 (15.0–31.2)	24.5 (18.4–31.3)
	Shot outcomes	Goals	28.2 (18.6–39.5)	19.0 (12.9–26.4)	13.0 (9.2–17.7)	14.5 (7.5–24.4)	22.3 (15.0–31.2)	14.7 (10.0–20.6)
		Saves	44.9 (34.3–63.0)	55.7 (47.1–64.5)	50.8 (43.1–59.6)	55.3 (36.1–78.1)	42.2 (33.5–65.8)	60.8 (47.8–75.2)
	Shot type	Hit	20.5 (12.2–31.2)	–	35.9 (30.1–42.0)	23.7 (14.7–34.8)	5.4 (2.0–11.3)	39.7 (30.4–50.9)
		Push/ Flick	25.6 (12.6–44.6)	69.0 (53.9–85.4)	12.9 (7.7–20.3)	27.6 (15.5–45.9)	75.0 (65.9–82.7)	23.3 (17.5–30.2)
		Sweep	6.4 (2.1–14.3)	5.6 (2.5–11.0)	13.0 (9.2–17.7)	6.6 (2.2–14.7)	–	3.8 (1.5–7.7)
		Reverse Hit	28.2 (18.6–39.5)	–	34.4 (28.6–40.4)	27.6 (18.0–39.1)	–	28.8 (22.4–35.9)
		Reverse Flick	2.6 (0.1–9.0)	16.2 (10.6–23.3)	1.1 (0.2–3.3)	2.6 (0.3–9.2)	11.6 (6.3–19.0)	1.1 (1.3–3.9)
		Deflection	16.7 (9.2–26.8)	8.5 (4.4–14.3)	2.7 (1.1–5.4)	9.2 (3.8–18.1)	6.3 (2.5–12.4)	3.3 (1.2–7.0)
	Shot Height	Below Shin Pad-Player	70.5 (59.1–80.3)	83.8 (76.7–89.4)	74.8 (69.1–80.0)	86.8 (77.1–93.5)	93.8 (87.5–97.5)	80.4 (74.0–85.9)
		Below Shin Pad-Goal	66.7 (55.1–76.9)	69.7 (61.5–77.1)	68.7 (62.7–74.2)	78.9 (68.1–87.5)	73.2 (64.0–81.1)	65.8 (58.4–72.6)
		Above Shin Pad-Player	20.5 (12.2–31.2)	12.7 (0.8–7.7–19.3)	21.8 (16.9–27.2)	5.3 (1.5–12.9)	0.9 (0.1–4.9)	3.3 (1.2–7.0)
		Above Shin Pad-Goal	29.5 (19.7–40.9)	29.6 (22.2–37.8)	30.5 (25.0–36.5)	13.2 (6.5–22.9)	21.4 (14.2–30.2)	17.9 (12.7–24.2)
		Unclear	0.1 (0.01–0.7)	–	–	–	5.4 (2.0–11.3)	15.8 (10.8–21.8)
	Players hit through	0	64.1 (52.4–74.7)	76.1 (68.2–82.8)	40.8 (34.8–47.1)	26.3 (16.9–37.7)	27.7 (19.6–36.9)	22.8 (16.9–29.6)
		1–2	34.6 (24.2–46.2)	23.2 (16.6–31.1)	55.7 (49.4–61.8)	69.7 (58.1–79.8)	61.6 (51.9–70.6)	62.0 (54.5–69.0)
		3–5	0.1 (0.01–0.7)	0.7 (0.02–3.9)	3.4 (1.6–6.4)	11.8 (5.6–21.3)	10.7 (5.7–18.0)	14.1 (9.4–20.0)
		6–8	–	–	–	–	–	–
Shooting Zone Entry	Shooting Zone Entry Type	Carried	26.9 (22.1–32.0)	39.8 (34.8–45.0)	29.1 (24.2–34.3)	38,7 (32.0–45.8)	64.0 (58.1–69.6)	39.2 (33.6–44.8)
		Hit	13.9 (10.3–18.1)	–	3.4 (1.7–5.9)	15.2 (10.4–24.5)	–	22.7 (18.1–27.7)
		Push	25.3 (20.4–30.6)	53.7 (48.2–60.1)	36.7 (30.4–44.3)	32.4 (26.0–39.2)	53.1 (47.2–59.0)	38.5 (33.1–44.2)
		Sweep	17.6 (13.6–22.2)	2.4 (1.1–4.6)	26.6 (21.9–31.7)	8.8 (5.3–13.6)	–	19.1 (14.9–23.9)
		Reverse Hit	0.5 (0.3–0.8)	–	0.1 (0.01–2.2)	7.4 (4.2–11.8)	–	4.9 (2.7–7.9)
		Deflection	0.2 (0.1–0.4)	3.3 (1.7–5.6)	0.9 (0.2–2.7)	3.9 (1.7–7.6)	2.4 (1.0–5.0)	1.0 (0.2–2.8)
		Reverse Flick	0.1 (0.01–0.2)	0.5 (0.01–1.9)	–	1.0 (0.1–3.5)	–	–
		Aerial	0.1 (0.02–0.3)	–	1.8 (0.7–4.0)	–	–	3.2 (1.6–5.9)
	Shooting Zone Entry Indoor	Right	27.8 (23.0–33.0)	8.1 (5.6–11.4)	36.1 (30.9–41.6)	41.7 (34.8–48.8)	36.7 (31.1–42.6)	49.2 (43.5–54.9)
		Middle	11.4 (0.8–15.4)	8.4 (5.8–11.7)	NA	18.1 (13.1–24.1)	19.2 (14.8–24.3)	–
		Left	19.8 (15.6–24.5)	18.2 (14.4–22.5)	33.9 (28.8–39.4)	29.4 (23.3–36.2)	37.4 (31.8–43.3)	49.5 (43.8–55.2)
		Outside 23	19.8 (15.6–24.5)	24.9 (20.6–29.7)	NA	7.8 (4.5–12.4)	4.9 (2.7–8.1)	–
	Shooting Zone entry Height	Below Shin Pad-Player	52.8 (47.2–58.3)	–	68.8 (63.5–73.8)	94.1 (90.0–96.9)	99.7 (98.1–99.9)	100.0
		Below Shin Pad-Goal	66.7 (61.2–71.8)	–	68.8 (63.5–73.8)	92.6 (88.1–95.8)	97.9 (95.5–99.2)	96.7 (94.1–98.4)
		Above Shin Pad-Player	18.5 (14.4–23.1)	–	2.4 (1.1–4.8)	4.9 (2.4–8.8)	0.3 (0.1–1.9)	0.0
		Above Shin Pad-Goal	0.5 (0.3–0.8)	–	2.1 (0.9–4.4)	6.4 (3.4–10.7)	1.7 (0.6–4.0)	3.2 (1.6–5.9)
	Players hit through	0	29.6 (24.7–34.9)	52.0 (46.8–57.2)	62.4 (56.9–67.7)	27.5 (21.5–34.1)	35.3 (29.8–41.2)	30.1 (25.0–35.5)
		1–2	31.5 (26.5–36.8)	7.6 (5.1–10.8)	8.3 (5.5–11.8)	59.3 (52.2–66.1)	55.9 (50.0–61.8)	54.4 (48.6–60.0)
		3–5	0.5 (0.3–0.8)	1.1 (0.3–2.8)	0.3 (0.01–1.7)	10.8 (6.9–15.9)	1.4 (0.4–3.5)	2.6 (1.1–5.0)
		6–8	0.1 (0.03–0.3)	–	–	–	–	–
Foul	Foul outcome	Penalty Corner	13.7 (10.3–17.9)	20.1 (15.1–25.8)	4.2 (1.7–8.5)	12.1 (9.2–15.6)	12.7 (9.3–16.8)	2.1 (0.7–4.9)
		Penalty Stroke	0.1 (0.01–0.2)	0.4 (0.01–2.4)	–	0.2 (0.01–13.2)	1.0 (0.2–2.6)	0.4 (0.1–2.4)
		Advantage	0.6 (0.4–0.9)	5.1 (2.7–8.8)	6.1 (2.9–10.9)	9.3 (6.7–12.4)	9.1 (6.2–12.7)	3.4 (1.5–6.6)
		Free Hit	79.8 (75.1–83.9)	74.4 (68.2 79.8)	89.1 (83.3–93.4)	78.1 (73.9–82.0)	77.0 (72.0–81.4)	94.0 (90.2–96.7)
	Foul sanction	Green	0.2 (0.1–0.4)	2.1 (0.7–4.9)	1.2 (0.1–4.3)	2.4 (1.1–4.3)	1.5 (0.5–3.5)	–
		Yellow	0.1 (0.03–0.3)	0.9 (0.1–3.1)	–	0.2 (0.01–13.2)	2.1 (0.1–4.3)	–
		Red	–	–	–	–	–	–

## Discussion

This is the first study to compare suspected injuries and game events across the three primary formats of field hockey. Although there was a trend towards higher rates of injury in men than women (specifically in Hockey5s for men and outdoor for women), there was no evidence of significant differences in suspected injury rates across the three formats, or between genders. Game events did differ significantly across formats of the game, however more events may not necessarily correspond with greater excitement, given the high proportion of unsuccessful attacking outcomes.

The rates of suspected injury reported in this study are higher than previous video analysis of outdoor hockey, however are not statistically different based on overlapping confidence intervals (8). This infers that the safety of the game has remained relatively consistent across almost 10 years (9), despite changes in rules including the removal of the ability of an outfield player to play with goal-keeping privileges, a potential increase in the use of aerial balls with greater contest allowed, and a perceived increasing use of skills such as drag flicks during penalty corners. Interestingly, despite the explicit concern of the International Hockey Federation regarding the potential danger of penalty corners (14), this event was responsible for just 11% of injuries (1 every 62 games). This implies that the penalty corner is not as dangerous as feared, that the use of protective equipment in this event reduces the risk of injury, or it may just reflect the trends seen in the sample of games included. Further research on a greater sample of penalty corners should be undertaken to explore player welfare in greater depth in this facet of the sport. Injuries to the head/face/neck area being the most commonly observed suspected injury in this study (26%: Table 1) is comparable with previous research which reported that injuries to the head/face accounted for 33% and 30% of injuries in women and men respectively (8). Furthermore, the majority of injuries being contusions (74%) also aligns with previous research (8) and also aligns with the findings related to ball-player contact being a key issue. The risk appears greater for defenders than attackers, who may put themselves in harm's way to prevent the progression of the ball. However, the high rate of penalization, but low level of sanctioning, would suggest that these ball-player contacts are accidental in nature. Combined, this suggest that these injuries are somewhat “expected” or “part of” the nature of the game.

There has been a large amount of anecdotal and social/ print media criticism regarding the safety and intended purpose of the Hockey5s format of the sport in recent years (15). In this study, there was no evidence of statistically significant differences in the rates of suspected injury between formats of play. This may be due to the small sample size used, as the rate ratios in the men's game specifically, suggests a 2.75-fold increased rate in Hockey5s compared to outdoor, while there was a 1.60-fold increased rate in Hockey5s compared to indoor. In the women's game, as only 1 suspected injury was noted in both indoor and Hockey5s, it is difficult to draw conclusions. In the men's game, the potentially relevant increase in rates, may be explained by the greater proportion of shots taken through players (59% in Hockey5s compared to 24% in indoor and 35% in outdoor; Table 2), and the proportion of shots above shin pad height (22% in Hockey5s, 21% in outdoor and 13% in indoor). This theory is supported in the women's data where outdoor had the highest injury rate and had the greatest proportion of shots through players (82%: vs. 76% in Hockey5s and 72% in indoor) and also had the most shots above the shin pad (5% vs. 1% in Hockey5s and 3% in indoor). Further, this also supports potential reasoning for a higher injury rate in men than women, although it was not statistically significant.

Although a greater number of game events was noted between the formats in both the men's and the women's games (Table 3), these do not appear to influence injury rates. In theory however, a greater level of specific game events may lead to higher levels of excitement within a game. A key goal of the International Hockey Federation is to “develop innovative and exciting entertainment events” (16). The concept of a more “exciting” sport is a difficult construct to research though. Excitement may relate to number of shots taken, number of entries into the shooting area, or number of counter attacks. In this study, the rates (per 10 min of play) for potentially “exciting” outcomes were highest in Hockey5s for men, while shots were highest in Hockey5s for women (Table 2). Despite this, these counterattacks did not necessarily lead to more excitement, with Hockey5s producing the lowest proportion of successful counter attacks outcomes in men. Furthermore, shot success was also lowest in Hockey5s for men and second lowest in women, compared to the other formats of the game. Goals scored is another proxy measure for excitement in games. The Hockey5s format had the greatest number of games which led to a final scoreline with a goal differential of five or more, compared to indoor and outdoor where the most common goal differential was two. This suggests uneven, or one-sided games, which may not have been “exciting” contests. There are two potential factors which contribute to this. Firstly, given the infancy of the format, the world rankings will take some time to reflect the true rankings of teams. Indeed, the World rankings for this format were only launched in March 2024 (17). Given the desire to increase participation in non-traditional hockey locations, all of the Continental Championships have been awarded the same weighting, therefore significant traditional “powerhouses” of the sport may be considerably lower than one might expect or be provided with similar points to more developing nations. This can lead to mismatches in competition, resulting in these high score differential outcomes, thus affecting the excitement of the game. The second reason for these differences again relates to the infancy of the sport and the need for teams and coaching staff to develop a more structured style of play and it may be some time before the tactical nuances we see in other formats will appear.

The primary limitation of this study was the low sample size, increasing in the likelihood of a type II error. A sample of 5 games from each format for both men and women were obtained, which, in the case of women's indoor and Hockey5s, only led to 1 observable suspected injury in each format. As this study relied on publicly available footage, further footage was not available. Furthermore, the generalizability of this data does not extend beyond the international game, and future evaluations should also consider different levels of player including amateur and youth. Given the public nature of the footage used, the research team had no control over the quality of the footage, the camera angles and the number of replays available, which have previously been identified as limitations in this type of study (11). Future work could aim to capture footage by the research team or work with broadcast companies to obtain multiple camera angles to allow for a more in-depth review of injuries. Despite using validated methods (6, 7), the use of a video-based suspected injury definition is also acknowledged as a limitation of the work. As outlined by Shill et al. (11), the use of medically diagnosed injury would significantly strengthen the study design, however no access to personal medical data was available in this study. The final limitation was that it was not possible, in some cases, to establish whether the removal of an injured player from the field was permanent or temporary.

## Conclusion and implications

This study demonstrated no evidence to suggest that injury is significantly higher in any hockey format, however this finding is limited by the low sample size. Initial trends suggest a potentially higher rate of injury in Hockey5s in the men's game, and within outdoor in the women's game. Hockey5s appears to have a higher number of more potentially “exciting” game events, however this does not necessarily translate to successful outcomes (i.e., more off target shots). Differences in team standard and a lack of tactical nuance are present at these early stages of this new format. Within outdoor, the rate of injury appears consistent, suggesting that rule changes and the development of tactics around the game have not negatively impacted player safety. The findings of this study suggest the need for further investigation with greater sample sizes, better measures of “excitement”, and a more in-depth assessment of penalty corners. Supporting such work with mixed methods approaches should be considered to understand fan engagement, player perceptions and injury risk.

## Data Availability

The raw data supporting the conclusions of this article will be made available by the corresponding author upon reasonable request.
