# Supplementary material for: Does the format matter? A cross-sectional analysis of suspected injuries and game events across the different versions of field hockey
Source: Front Sports Act Living. 2025 Sep 2;7:1565036. doi: 10.3389/fspor.2025.1565036 (PMC12441797; doi:10.3389/fspor.2025.1565036)

| Table S1: Rules of different hockey formats (FIH, 2024) | | | |
| --- | --- | --- | --- |
| Rules | Outdoor Hockey | Indoor Hockey | Hockey5s |
| No. of players per team | 11 | 6 | 5 |
| No. of substitutes | 5 | 6 | 4 |
| Match duration | 60 minutes  (4 quarters) | 40 minutes  (4 quarters) | 20-30 minutes  (2 halves) |
| Pitch dimensions | 91.4m x 55m | 44-36m x 22-18m | 55-40m x 42-28m |
| Boundary height | No Boundary boards | 10cm | 10cm – 25cm |
| Permitting scoring zones | Within the attacking circle (14.6m from goal) | Within the attacking circle (9-11m from goal) | Within attacking half (27.5-20m from goal) |
| Restrictions on pass and shot type | No restrictions | Ball must not be lifted. | No restrictions |

Table S2: Injury rates and rate ratios by format and sex.

| Sex | Format | Injuries per game | Rate / 1000 hours | Rate Ratio |
| --- | --- | --- | --- | --- |
| Men | Outdoor | 1.6 | 72.7 (31.4-143.3) | 1.0 |
|  | Indoor | 1.0 | 125.0 (40.6-291.7) | 1.72 (0.44-5.96); p=0.35 |
|  | Hockey5s | 1.0 | 200.0 (64.9-466.7) | 2.75 (0.71-9.53); p=0.10 |
|  | Indoor (ref) vs 5s |  |  | 1.60 (0.37-6.96); 0.47 |
| Women | Outdoor | 1.4 | 63.6 (25.6-131.1) | 1.0 |
|  | Indoor | 0.2 | 25.0 (6.3-139.3) | 0.39 (0.01-3.06); 0.41 |
|  | Hockey5s | 0.2 | 40.0 (1.0-222.9) | 0.62 (0.01-4.89); 0.74 |
|  | Indoor (ref) vs 5s |  |  | 1.60 (0.02-125.59); 0.77 |
| Men/ Women Comparisons | Outdoor | NA | NA | 1.14 (0.36-3.70); 0.80 |
|  | Indoor | NA | NA | 5.00 (0.56-236.49); 0.13 |
|  | Hockey5s | NA | NA | 5.00 (0.56-236.49); 0.13 |

Appendix A: Variable Definitions

**MATCH EVENTS**

Play starts.

- The start of a quarter in field hockey and indoor hockey and the start of a half in Hockey 5s

Play Ends

- The end of a quarter in field hockey and indoor hockey and the end of a half in Hockey 5s

Tackle

- A defender taking the ball from an attacker in open play.

Turnover

- Loss of ball possession to opposing team

Counter-attack.

- A turnover that leads to a shooting zone entry
  - Successful counterattack = a shot on target or penalty corner within the same phase of play.
  - Unsuccessful – circle entry with no shot or pc

Shooting zone entry

- Entering a zone where you can legally shoot and score (circle for indoor and field, attacking half for 5's).

Pass

- A player transfers the ball to the intended target (the stick of another player on their team)
  - Successful 🡪 the ball reaches the intended target.
  - Unsuccessful 🡪 the ball does not reach the intended target.

Interception

- Catching a pass made by a player on the opposite team.

Mistrap

- When the ball has reached the intended player but said player is unable to keep the ball under control and loses possession

**FOUL**

Penalty Challenge (Hockey 5s)

- For an offence by a defender within the half of the court they are defending which prevents the probable scoring of a goal
  - One attacking player starts with the ball on the quarter line, with all others behind the halfway line except from goalkeeper. When the whistle blows, the attacking player must travel 4 meters before attempting a shot, once ball has been played, players on center line may re-join play.

Penalty corner (Field & Indoor)

- For an offence by a defender in the circle which does not prevent the probable scoring of a goal
  - The attacking player taking the push must have 1 foot outside the pitch on the penalty corner attacker’s mark. On the umpire’s command, they play it to their teammates at the top of the circle where it must leave the circle before a shot on goal can be attempted. Not more than 5 players incl. the keeper may be positioned behind the backline.

Penalty Stroke (Field & Indoor)

- For an offence by a defender in the circle which prevents the probable scoring of a goal
  - The attacker stands behind the ball on the penalty spot with the goalkeeper positioned on the goal line, not moving until the attacker either pushes, flicks or scoops the ball towards goal. All other players must be a set distance away, depending on the format of hockey.

Penalty Stroke (5s)

- For an action preventing a certain goal (such as a foot on the goal line)
  - For a deliberate foul by the goalkeeper during a challenge

Advantage

- When there is a foul in play, but the referee plays advantage.

Free hit

- The ball is stopped and played by the awarded team where the foul was blown up.

Green (card)

- A player is warned and temporarily suspended for 2 minutes of playing time.

Yellow (card)

- A player is temporarily suspended for a minimum of 5 minutes of playing time.

Red (card)

- A player is permanently suspended from the current match.

Suspected injury

- A player staying down or demonstrating physical discomfort (e.g., limping, slow to return to feet, clutching at area in pain, seeking medical attention)

**SHOOTING**

Ball into D

- Any time the ball is passed into the D.

Shot

- Any attempt made by a player to score a goal (from a position they can legally score a goal)

On target

- Any shot attempt that results in either a save (goalie or defensive) or a goal.

Off target

- Any shot attempt that goes wide or over the goal.

Goal

- The ball crosses the goal line into the net.

Save

- The defensive player prevents the ball entering the goal.

Rebound

- When the ball bounces off a keeper or rebound board to remain in play.

**TYPE**

Hit

- Striking the ball by swinging the stick through the air with a back swing and follow through.

Push

- Moving the ball by placing the stick in directly in contact with the ball then pushing away from or across the body along the ground.

Sweep

- A hit with both hands at the top of the grip but with the back swing and follow through mostly on the ground.

Reverse hit

- A low horizontal hit with both hands at the top of the grip on the left-hand side.

Flick

- Raising the ball off the ground with the stick further than player space and overhead height using a push like action

Ariel

- A form of pass where a ball is lifted above head height from one player to another on the same team.

Deflection

- The act of redirecting the course of a ball hit by someone on the same team

Flat

- When the ball remains in contact with the pitch surface during entire journey from one player to another.

Lifted Pass

- When the ball is not in contact with the pitch surface but remains below knee height during the journey from one player to another.

**DEFENDER’S ACTIONS**

Evasive Action

- A player moves out of the way of a shot/pass with no regard to block the shot.

High Risk Action

- A player puts themselves in the path of the ball / moves towards the ball.

No Action

- A player leaves their stick in the way if a shot or does not take evasive action or high-risk action.

Wrong side of the shot

- Where the defender must reach across the opposition player to attempt a tackle

**ATTACKER’S ACTIONS**

Dangerous wind-up

- Swinging within a sticks’ length of another player.

Dangerous follow through

- Following through within a sticks’ length of another player.

**HEIGHT**

Safe Height

- Ball passes defender at knee level or below.

‘Dangerous’ Height

- Ball passes defender above the knee in close proximity to a player(s)

**HITTING THROUGH PLAYERS**

Players at Risk of Being Hit

- Anybody at a reasonable risk of being hit from a shot (providing the shot were to go in intended direction)

**GOAL DIFFERENTIAL**

- The difference in number of goals between teams

Appendix B:


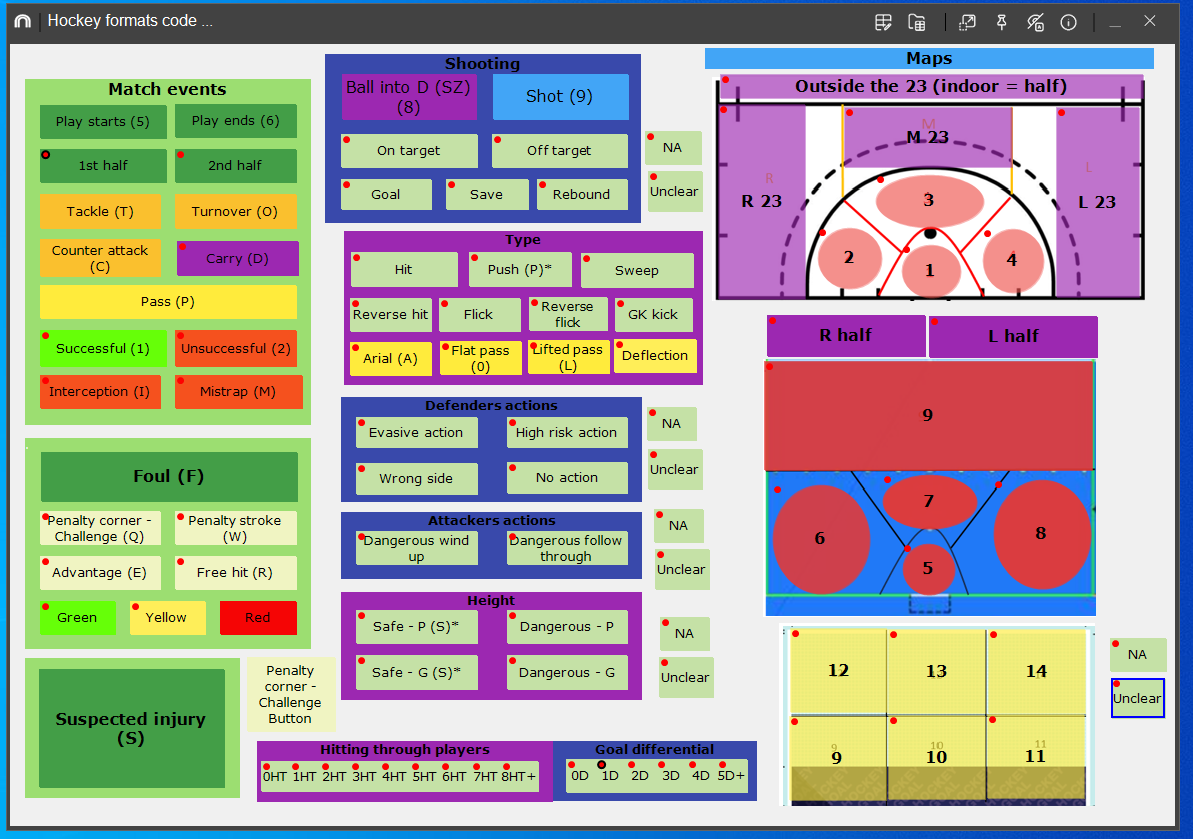

Supplement: Supplementary file 1 [file Supplementaryfile1.docx]
